# Circadian Rhythms and Mitochondria: Connecting the Dots

**DOI:** 10.3389/fgene.2018.00452

**Published:** 2018-10-08

**Authors:** Laura Sardon Puig, Miriam Valera-Alberni, Carles Cantó, Nicolas J. Pillon

**Affiliations:** ^1^Department of Molecular Medicine and Surgery, Karolinska Institutet, Stockholm, Sweden; ^2^Nestlé Institute of Health Sciences, Lausanne, Switzerland; ^3^School of Life Sciences, Ecole Polytechnique Fédérale Lausanne, Lausanne, Switzerland; ^4^Department of Physiology and Pharmacology, Karolinska Institutet, Stockholm, Sweden

**Keywords:** circadian rhythm, mitochondria dynamics, metabolism, acetylation, AMPK, HIF1α

## Abstract

Circadian rhythms provide a selective advantage by anticipating organismal nutrient needs and guaranteeing optimal metabolic capacity during active hours. Impairment of circadian rhythms is associated with increased risk of type 2 diabetes and emerging evidence suggests that metabolic diseases are linked to perturbed clock machinery. The circadian clock regulates many transcriptional–translational processes influencing whole cell metabolism and particularly mitochondrial activity. In this review, we survey the current literature related to cross-talks between mitochondria and the circadian clock and unravel putative molecular links. Understanding the mechanisms that link metabolism and circadian responses to transcriptional modifications will provide valuable insights toward innovative therapeutic strategies to combat the development of metabolic disease.

## Introduction

The circadian clock is present in most organisms on Earth. In mammals, the circadian clock orchestrates 24 h oscillations in many physiological processes. Blood pressure, body temperature, and hormonal secretion, among others, follow daily fluctuations according to the rotation of the Earth (Douma and Gumz, 2018). These rhythms are driven by light information sensed by the eye and processed in the central clock situated in the suprachiasmatic nucleus (SCN). However, peripheral tissues have their own clocks and also exhibit independent circadian oscillations, synchronized to the central clock through nervous and hormonal signals. The molecular machinery of the clock is a transcriptional feedback loop similar in all cells (Buhr and Takahashi, 2013). The transcription factors CLOCK (clock circadian regulator) and BMAL1 (ARNTL, aryl hydrocarbon receptor nuclear translocator like) form the main activator heterodimers that promote the expression of the repressor proteins period circadian regulator (PER) and cryptochrome circadian regulator (CRY). These two proteins later dimerize and inhibit CLOCK:BMAL1 activity. Similarly, CLOCK:BMAL1 dimers promote the transcription of DBP (D-Box Binding PAR BZIP transcription factor), which enhances the activity of PER:CRY (Yamaguchi et al., 2000). CLOCK:BMAL also promotes the expression of the orphan nuclear-receptor REVERBα (NR1D1, nuclear receptor subfamily 1 group D member 1), that negatively regulates BMAL1 transcription (Solt et al., 2011; Buhr and Takahashi, 2013). RORα (retinoic acid receptor-related orphan receptors), a BMAL1 transcription activator, competes with REVERBα for BMAL1 binding elements (Solt et al., 2011). All these elements can be regulated by transcription and post-translational modifications and in turn trigger multiple responses in the cells.

Both the central and peripheral clocks can be affected by non-light factors such as metabolic changes or physical activity and are therefore under scrutiny for their involvement in metabolic diseases (Mohawk et al., 2012). In fact, diets high in fat and sugar and sedentary lifestyle are associated with disturbances in circadian rhythms. After only 1 week on high fat diet, mice present a lengthened circadian period compared to their chow fed littermates, independently of body weight change (Kohsaka et al., 2007). Conversely, dysregulation of the circadian clock has a critical impact on cellular metabolism. For example, REVERBα deficient mice present a shortened oscillation period compared to wild-type mice (Han et al., 2008). Mice with liver-specific of BMAL1 suffer from metabolic complications, including hypoglycemia in the post-absorptive phase (Lamia et al., 2008) and the inactivation of CRY1 and CRY2 leads to fasting hyperglycemia (Zhang et al., 2010). In humans, night-shift work is associated with an increased risk of obesity and type 2 diabetes and their consequent co-morbidities and mortality (van Drongelen et al., 2011; Rajaratnam et al., 2013). Overall, these observations suggest an intimate and reciprocal relationship between the metabolic status of the cell/ organism and the circadian clock.

Mitochondria are one of the major cellular nodes for nutrient integration and ATP generation. Mitochondria are therefore highly dynamic, and their activities change according to the cell nutritional status at different times of the day. This flexibility can be achieved through diverse mechanisms, including specific post-translational modification events and major changes in mitochondrial bioenergetic profiles through fusion and fission events. In addition, mitochondria communicate with other organelles in the cell, including the nucleus, by generating ATP and releasing metabolites, peptides, and even nucleic acid fragments (Kohsaka et al., 2014). Because mitochondria are central to metabolic integration and can regulate transcription mechanisms, it is likely that one or several mechanisms link mitochondrial function to circadian rhythms. In the following sections, we will describe a couple of possible cross-talk mechanisms between mitochondrial function and the circadian clock.

## Possible Mechanisms Linking the Clock and Mitochondrial Function

### Mitochondrial Dynamics

Initial observations on the relationship between the clock and mitochondrial morphology were made more than 20 years ago on rat hepatocytes, where mitochondrial tubular structure was significantly diminished upon transition from the light to the dark phase (Uchiyama, 1981). Mitochondrial dynamics is the process by which mitochondria continuously fuse and divide. This allows the cellular mitochondrial network to modulate their bioenergetic properties according to the nutritional demands of the organism, regulate mitochondrial motility and removal of damaged mitochondria. Experiments in cultured fibroblasts, myocytes, and mouse primary adipocytes demonstrate that mitochondrial fusion, driven by the GTPases mitofusin 1 and 2 (MFN1 and MFN2) and optic atrophy 1 (OPA1), is linked to a higher efficiency in the coupling of substrate oxidation with ATP production (Gomes et al., 2011). This has been proposed to rely on increased levels of dimerization and activity of ATP synthase upon mitochondrial elongation (Gomes et al., 2011). Conversely, mitochondrial fragmentation, controlled by the GTPase dynamin-related protein 1 (DRP1), is correlated to lower coupling efficiency. As an example, brown adipocytes require mitochondrial fission in order to maximize their uncoupling capacity in response to adrenergic stimuli (Bach et al., 2003).

Nutrient availability and metabolic demands impact on mitochondrial architecture: elongation is often observed in response to nutrient deprivation, allowing efficient ATP production (Gomes et al., 2011). On the contrary, mitochondrial fission is commonly observed upon nutrient overload (Bach et al., 2003). These changes can be controlled through acute and long-term mechanisms. In an acute fashion, multiple signaling paths can impinge on the activity of the mitochondrial fission machinery. For example, protein kinase A (PKA) activation during fasting or after adrenergic stimulation can phosphorylate DRP1, leading to enhanced (Han et al., 2008; Wang W. et al., 2012; Wikstrom et al., 2014) or decreased (Chang and Blackstone, 2007; Cribbs and Strack, 2007; Cereghetti et al., 2008) fission rates in a tissue-specific fashion. Given that PKA activity is controlled by multiple hormones, this ensures a quick, tight coupling of mitochondrial architecture to the organismal metabolic state. In chronic situations of nutrient overload mitochondrial look rounder and smaller and this has been related to the transcriptional repression of the MFN2 gene (Bach et al., 2003). Therefore, mitochondrial architecture can be regulated both at the transcriptional and post-transcriptional levels.

In cultured fibroblasts, the morphology of the mitochondrial network exhibits circadian rhythmicity, changing from a tubular mitochondrial network at 16 h after serum shock to highly fragmented network at 28 h post-synchronization and matching the rhythmicity of ATP content and oxidative phosphorylation (Schmitt et al., 2018). These rhythms are lost in fibroblasts from PER1/PER2 mutant mice, which lack a functional circadian clock. Under these circumstances, ATP levels do not cycle and the mitochondrial network remains fragmented at all time points, testifying to perturbed mitochondrial dynamics (Schmitt et al., 2018). Further, mice lacking PER1/2 present altered oscillations in mitochondrial respiration (Neufeld-Cohen et al., 2016). Similarly, cultured hepatocytes from liver-specific BMAL1 deficient mice present swollen mitochondria, correlated to reduced levels of the mitophagy proteins Fis1 and Pink1, as well as an inability to adapt to different nutrient conditions (Jacobi et al., 2015). However, whether the changes in mitochondrial morphology are causal or consequential to metabolic changes in the cells has not been fully elucidated. Nevertheless, BMAL1^-/-^ mice display decreased levels of mitochondrial fusion proteins MFN1 and OPA1 in the heart (Kohsaka et al., 2014), suggesting a potential direct control of the clock on mitochondrial architecture.

Changes in mitochondrial architecture might also influence circadian rhythms. Upon serum-shock on DRP1 deficient mouse embryonic fibroblasts (MEFs), BMAL1 and PER1/2 lose their circadian variation, and cells lacking DRP1 do not display ATP oscillations. Consistent with these observations in cultured cells, circadian ATP production is eliminated in DRP1-deficient mice hippocampi, indicating that the circadian activation of DRP1 plays a central role in coupling circadian and mitochondrial metabolic cycles (Schmitt et al., 2018).

Disruption of mitochondrial dynamics at the whole body level compromises organismal viability. Nevertheless, model organisms with tissue specific deletions of MFN1 teach us that impaired mitochondrial fusion compromises the ability of tissues to shift between lipid and carbohydrate energy sources, tipping the balance toward higher lipid use (Kulkarni et al., 2016; Ramírez et al., 2017). These observations further support that mitochondrial dynamics are a critical effector mechanism for the regulation of energy substrate utilization in response to daily cycles.

### Deacetylation Mechanisms and the Clock

In order to maintain synchrony with the environment, the clock machinery interacts with other cell mechanisms, one of them being the nicotinamide adenine dinucleotide (NAD^+^)-dependent protein deacetylases sirtuins, which by sensing the cellular metabolic state, provide flexibility to the clock. NAD^+^ is a central metabolic cofactor that regulates cellular metabolism and energy homeostasis, notably through the activation of sirtuins. Mammals have seven sirtuins (SIRT1–7) which are found in different subcellular locations, with SIRT6 and SIRT7 being exclusively present in the nucleus, while SIRT3, SIRT4, and SIRT5 are confined to mitochondria (Vassilopoulos et al., 2011). SIRT1, which can be found in cytosolic and nuclear compartments (Tanno et al., 2007), has been extensively studied in the context of metabolic regulation. In particular, SIRT1 participates in the transcriptional adaptation to nutrient deprivation by deacetylating a constellation of transcriptional coregulators, transcription factors and nuclear receptors that control mitochondrial biogenesis and fatty acid oxidation-related gene expression (Nakahata et al., 2007, 2008; Scarpulla, 2011). In response to increasing NAD^+^ levels, SIRT1 causes the deacetylation of PER2, leading to its degradation (Asher et al., 2008). BMAL1 may also be a target for SIRT1 deacetylation, which would prevent the repressor action of CRY on CLOCK/BMAL1 transcriptional activity (Nakahata et al., 2008). Thus, there appears to be a direct connection between nutrient and energy stress and circadian rhythmicity. Interestingly, CLOCK is by itself an acetyl transferase enzyme (Kohsaka et al., 2014), which hints the possibility that CLOCK and SIRT1 could counteract on common targets.

The clock machinery controls the gene expression of nicotinamide phosphoribosyltransferase (NAMPT), a key enzyme for the synthesis of NAD^+^ from nicotinamide (Ramsey et al., 2009). Upon SIRT1 activation, CLOCK:BMAL1 directly binds the NAMPT promoter, prompting its transcription. This allows sustained NAD^+^ levels to maintain SIRT1 activity during non-feeding times. The increase in NAD^+^ triggered by the transcriptional regulation of NAMPT can potentially impact on other members of the sirtuin family. Thus, NAD^+^ serves as a metabolic link between circadian clocks and mitochondrial function through a NAD^+^ and sirtuin-dependent deacetylation (Ramsey et al., 2009). Several NAD^+^ precursors, such as nicotinamide riboside, are currently being used as a dietary supplementation strategy to prevent metabolic and age-related complications. No changes in circadian behavior have been observed in mice treated with nicotinamide riboside, probably because the compound was administered with the diet (Kohsaka et al., 2014). Nevertheless, it should be noted that nicotinamide riboside metabolizing enzymes are expressed in a circadian pattern, with higher expression during feeding periods (Kohsaka et al., 2014).

SIRT3, a mitochondrial sirtuin, tightly controls mitochondrial oxygen consumption rate (Lakso et al., 1996), and mediates inner mitochondrial membrane fusion by deacetylating OPA1 (Samant et al., 2014). Under stress conditions, such as aortic constriction, OPA1 is hyperacetylated in mice cardiac muscle, which compromises its GTPase activity and leads to mitochondrial fission (Samant et al., 2014). Deacetylation of OPA1 by SIRT3 restores its GTPase activity (Samant et al., 2014), preserving the mitochondrial network and protecting cardiomyocytes from cell death (Samant et al., 2014). These results suggest that during nutrient deprivation periods, when NAD^+^ rises, SIRT3 contributes to mitochondrial elongation via the deacetylation of OPA1, hence tying together the activities of the clock, sirtuins, and mitochondrial architecture enzymes.

SIRT6 function is also linked to the regulation of oxidative and glycolytic metabolism. SIRT6 deacetylates H3K9 at the promoters of several key glycolytic genes (Zhong et al., 2010). Consequently, reduced SIRT6 activity increases the acetylation and transcription rates of glycolytic genes, leading to enhanced glucose uptake and anaerobic glycolysis, to the point that SIRT6 deficient mice die from hypoglycemia shortly after birth due to abnormally high glucose uptake rates in skeletal muscle and brown adipose tissue (Mostoslavsky et al., 2006; Zhong et al., 2010). SIRT6 has been demonstrated to regulate the hepatic circadian clock by controlling the recruitment to chromatin of CLOCK:BMAL1, as well as SREBP-1, and this results in the cyclic regulation of hepatic metabolism related to fatty acid and cholesterol metabolism (Masri et al., 2014).

As a whole, these observations suggest that NAD^+^ availability and sirtuins determine the function of the peripheral clock, providing an exquisite mechanism to fine-tune the metabolic status of the cell to circadian rhythmicity. In turn, CLOCK-driven NAD^+^ biosynthesis allows sustained sirtuin activity, impacting on nutrient handling and mitochondrial activity.

### AMPK Activation and the Clock

There is substantial evidence that adenosine monophosphate-activated protein kinase (AMPK) is involved, and even directly regulates clock mechanisms. AMPK is a serine/threonine kinase activated by increased AMP/ATP ratio (Xue and Kahn, 2006) and plays a central role in the regulation of cellular energy balance and the ability of cells to adapt fuel uptake and preference to nutrient availability. Activation of AMPK by AMP represses anabolic pathways and promotes catabolic ATP-producing mechanisms such as glucose uptake, glycogen breakdown, and expression of nutrient transporters (Xue and Kahn, 2006). At the same time, AMPK promotes mitochondrial biogenesis by increasing NAD^+^ levels and triggering the sirtuin/PGC1α axis (Cantó et al., 2009).

Initial evidence for a direct link between AMPK activation and the circadian clock machinery came from metformin-treated fibroblasts which exhibit a shortened daily cycle by 1 h. This is mechanistically explained by AMPK driving the phosphorylation of casein kinase Iε (CKIε), which then phosphorylates PER2 and facilitates its degradation (Um et al., 2007). AMPK directly phosphorylates the core clock protein CRY1 (Lamia et al., 2009): the AMPK activator AICAR reduces the half-life of CRY1 and enhances the amplitude of circadian expression of REVERBα and DBP (Lamia et al., 2009). CRY protein stability is crucial for defining the pattern of expression of mammalian clock genes (Zhang et al., 2010) and consequently, the destabilization of CRY1 by AMPK affects the overall clock gene expression, notably by promoting CLOCK and BMAL1 expression (Lamia et al., 2009). Indeed, AICAR-induced AMPK activation in fibroblasts induces a phase shift of BMAL1 (Kim et al., 2016). This is potentially part of an important metabolic cross-talk, since AICAR-stimulated glucose uptake in skeletal muscle is reduced in BMAL1 deficient mice (Harfmann et al., 2016). These results suggest that some of the metabolic effect of AMPK might be mediated through regulation of the clock.

AMPK triggers mitochondrial fission upon energetic stress by phosphorylating the kinase unc-51 like autophagy activating kinase 1 (ULK1) and the mitochondrial fission factor (MFF), favoring the recruitment of DRP1 and eventually enhancing fission rates in order to maximize mitophagic processes on damaged mitochondria (Toyama et al., 2016). Additionally, the MFN*2* gene is controlled by AMPK in mouse skeletal muscle during fasting or in response to contraction (Cantó et al., 2010). The transcriptional increase in MFN2 might explain the more elongated mitochondrial network observed in mouse models expressing the R225Q form of AMPKγ3, which leads to higher basal AMPK activity (Garcia-Roves et al., 2008). AMPK therefore has a dual effect on mitochondrial dynamics. AMPK activation can promote rapid fission to remove dysfunctional mitochondrial elements and rapidly obtain energy from mitophagy products and also lead to long-term synthesis of new mitochondria and increased fusion to maximize ATP generation efficiency upon future challenges.

Mitochondria generate energy by converting lipids and carbohydrates into ATP through oxidative phosphorylation (de Goede et al., 2018), therefore controlling energy levels. NAD^+^, ATP, and glucose are then sensed by AMPK, therefore providing a direct route from mitochondrial energy production to the modulation of transcription and clock gene expression.

### Oxygen, ROS, HIF1α, and the Clock

Mitochondrial respiration in skeletal muscle oscillates (van Moorsel et al., 2016), probably as a consequence of changes in whole body metabolic status. Indeed, the ability to adjust fuel utilization to nutrient availability allows tissues to switch from carbohydrates during feeding to fatty acids during fasting. This is reflected by indirect calorimetry showing lower carbohydrate oxidation and respiratory exchange ratio in the fed state (van Moorsel et al., 2016). Tissue oxygen levels exhibit daily oscillations with increased oxygen levels during the dark phase, when rodents are active (Adamovich et al., 2017). *In vitro*, inducing 12-h cycles from 5 to 8% oxygen is sufficient to synchronize the cells and induce cycling of the core clock genes (Adamovich et al., 2017). This phenomenon is dependent on hypoxia-inducible factor 1-α (HIF1α) which in turn regulates the rhythmicity of several core clock genes by regulating transcription (Wu et al., 2017). Additional studies have suggested that HIF1α directly binds to the PER2 and CRY1 promoters (Bhatti et al., 2017; Peek et al., 2017), providing a possible link between oxygen levels and the regulation of peripheral clocks. Conversely, deletion of BMAL1 in skeletal muscle cells and fibroblasts impairs mitochondrial respiration and increases the levels of HIF1α under hypoxic conditions (Peek et al., 2017). This suggests a bi-directional regulation of HIF1α and clock genes to maintain proper mitochondrial function (Peek et al., 2017).

Oxidative phosphorylation at the mitochondrial membrane is a major source of cellular reactive oxygen species (ROS), it is therefore not surprising that circadian rhythms in the redox state are also present. The most ancient redox rhythm is the one of peroxiredoxin, antioxidant proteins which oxidized/reduced state oscillates in archae, bacteria, and eukaryotes (O’Neill and Reddy, 2011; Edgar et al., 2012). Other antioxidant systems have been shown to follow circadian rhythms, such as glutathionylation in the SCN that peaks at night and is lower during the day, indicating a relatively oxidized state during the active/feeding period (Wang T.A. et al., 2012). Oxidative stress is damaging and must be compensated by several antioxidant mechanisms such as enzymes and glutathione. An imbalance between ROS production and antioxidant defenses leads to pathologies and is typically observed in cells and animal models of metabolic diseases. A couple of *in vitro* studies suggest a cross-talk between ROS production and clock gene regulation (Sundar et al., 2018). Activation of macrophages with lipopolysaccharides disrupts both the phase and amplitude of cycling of PER2, a process dependent of the production of ROS (Wang et al., 2016), since addition of antioxidants to the cells restored a proper rhythm. Conversely, macrophages from BMAL1 deficient mice have impaired NO production (Wang et al., 2016), suggesting that clock transcription factors regulate redox processes. Indeed, CLOCK and BMAL1 directly regulate NRF2 (Pekovic-Vaughan et al., 2014), a critical transcription factor responsible for the production of most antioxidant defenses. Altogether, there is evidence for a redox/clock cross-talk but the exact mechanisms and whether it is regulated by the metabolic status of the cells is unknown.

## Conclusion and Research Perspectives

Human studies are showing that night-shift work is associated with increased risk of metabolic disorders and that obese individuals present altered biological rhythms (Itani et al., 2011; van Drongelen et al., 2011; Canuto et al., 2014). In addition, industrialized societies display very erratic feeding patterns (Gill and Panda, 2015), which compromises circadian entrainment. Given the strong influence of circadian rhythms on whole body metabolic homeostasis, the above observations have major implications for global heath and might contribute to the increasing rates of obesity and metabolic disorders worldwide (Visscher et al., 2015). Time of feeding and composition of meals might affect both the central and peripheral clocks and therefore may be an interesting approach to impact mitochondrial function and normalize clock and metabolic processes. While mouse experiments demonstrate that time-restricted feeding can prevent metabolic disorders (Gill and Panda, 2015), such lifestyle interventions are difficult to apply in humans. Therefore, there is an urge to discover new strategies to entrain the clock in situations of metabolic disease. For this, a better understanding of the link between our internal clock and metabolism is needed.

**FIGURE 1:**
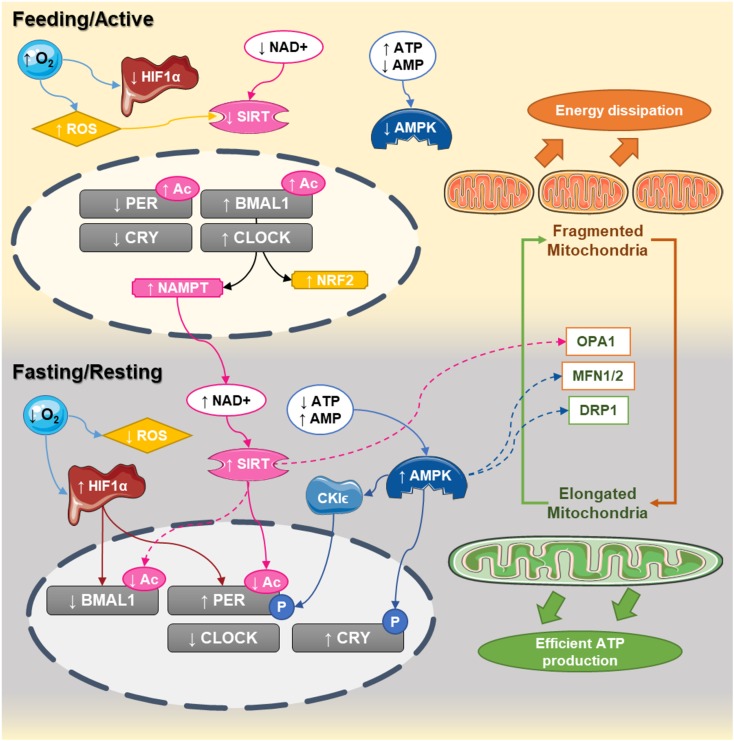
Circadian interactions between the clock and mitochondrial dynamics. In humans, CLOCK:BMAL1 dimers peak at the end of the day (active phase) while PER:CRY are elevated at the end of the dark phase. During the active phase, nutrient availability decreases NAD^+^ levels and increases ATP content, decreasing the activity of sirtuins and AMPK, respectively. At the same time, the elevated oxygen content inhibits HIF1α and increases reactive oxygen species production. During that phase, the central clock promotes the transcription of NAMPT and NRF2, which will progressively build up the levels NAD^+^ and antioxidant defenses. At the end of the active phase and during the fasting phase, NAD^+^ levels are higher, while ATP levels decrease. This leads to activation of sirtuins and AMPK and the promotion of mitochondrial fusion. Activation of sirtuins deacetylates the core clock components, while the decreased oxygen levels promote the activation of HIF1 which bind to the promoters and activates the clock genes. Elongated mitochondria promote coupling and efficient ATP production, starting a new cycle.

In this review, we have suggested several mechanisms by which mitochondria and energy metabolism could be involved in the disruption of the circadian clock and vice versa. Glucose and lipid metabolism can feed back on the core clock and therefore, nutrients appear to be crucial regulators of the circadian clock (**Figure 1**). Functional defects in mitochondria have been implicated in the pathophysiology of metabolic disorders, including type 2 diabetes, obesity, dyslipidemia, and cardiovascular diseases (Bhatti et al., 2017) and experimental data support the idea that mitochondrial function is tightly linked to the clock machinery. The circadian nature of mitochondrial morphology through fusion/fission mechanisms and its relation to metabolic rhythms and clock regulation is the subject of intense research (Manella and Asher, 2016) and might open new therapeutic perspectives. Further studies on the circadian nature of mitochondrial biology will shed light on the underlying mechanisms linking daily rhythms and energy metabolism. It will also help us understanding whether nutrients and mitochondrial function might be able to engage circadian rhythmicity in obese and diabetic patients as well as in populations at risk. In this sense, mouse experiments support that feeding patterns can engage daily circadian rhythm even in situations of disrupted clock gene expression (Gill and Panda, 2015). It will be important to evaluate whether this also occurs in humans, given that human lifestyles and daily behavior patterns are influenced by multiple social aspects beyond feeding. Nevertheless, this opens potential avenues for the development of interventional strategies for the prevention or the treatment of metabolic disorders.

## Author Contributions

LSP and MV-A contributed equally to the collection of references and writing of the manuscript. CC and NP contributed equally to the writing, review, editing, and supervision. All authors approved the final version to be published.

## Conflict of Interest Statement

MV-A and CC are employees of the Nestlé Institute of Health Sciences.The remaining authors declare that the research was conducted in the absence of any commercial or financial relationships that could be construed as a potential conflict of interest.
